# Tricyclic Pyrazole-Based Compounds as Useful Scaffolds for Cannabinoid CB_1_/CB_2_ Receptor Interaction

**DOI:** 10.3390/molecules26082126

**Published:** 2021-04-07

**Authors:** Battistina Asproni, Gabriele Murineddu, Paola Corona, Gérard A. Pinna

**Affiliations:** Department of Chemistry and Pharmacy, University of Sassari, Via Muroni 23/A, 07100 Sassari, Italy; muri@uniss.it (G.M.); pcorona@uniss.it (P.C.); pinger@uniss.it (G.A.P.)

**Keywords:** tricyclic pyrazoles, structure-activity relationship, CB_2_ agonist, CB_1_ agonist, CB_1_ neutral antagonist, anti-nociceptive activity, glaucoma, obesity

## Abstract

Cannabinoids comprise different classes of compounds, which aroused interest in recent years because of their several pharmacological properties. Such properties include analgesic activity, bodyweight reduction, the antiemetic effect, the reduction of intraocular pressure and many others, which appear correlated to the affinity of cannabinoids towards CB_1_ and/or CB_2_ receptors. Within the search aiming to identify novel chemical scaffolds for cannabinoid receptor interaction, the CB_1_ antagonist/inverse agonist pyrazole-based derivative rimonabant has been modified, giving rise to several tricyclic pyrazole-based compounds, most of which endowed of high affinity and selectivity for CB_1_ or CB_2_ receptors. The aim of this review is to present the synthesis and summarize the SAR study of such tricyclic pyrazole-based compounds, evidencing, for some derivatives, their potential in the treatment of neuropathic pain, obesity or in the management of glaucoma.

## 1. Introduction

**Figure 1:**
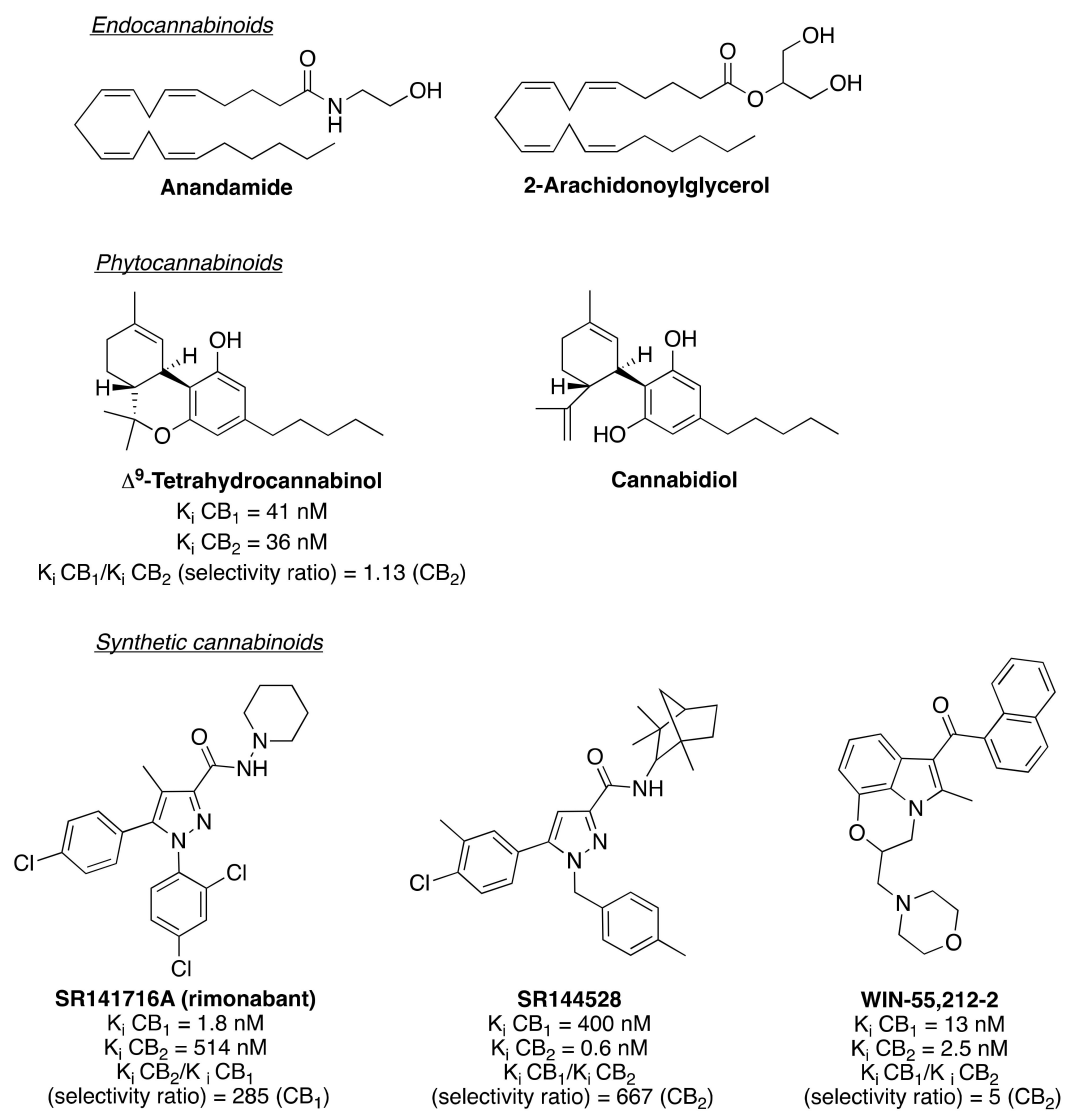
Chemical structure of representative cannabinoids.

Cannabinoids are a class of different chemical compounds (Figure 1), including the endocannabinoids (produced naturally in the body by humans and animals such as anandamide and 2-arachidonoylglycerol), the phytocannabinoids (derived from *Cannabis*, exemplified by Δ^9^-tetrahydrocannabinol, the major psychoactive component of *Cannabis sativa*, commonly known as marijuana), and the synthetic cannabinoids (produced chemically by human, comprising a wide array of chemical entities, i.e., the pyrazole-based compounds SR141716A and SR144528 and the indole based compound WIN-55,212-2) [1,2,3]. The physiological and behavioral effects of cannabinoids appear directly correlated to their affinity towards two different classes of specific receptors: CB_1_ receptors located predominantly in the central nervous system [4], and CB_2_ receptors which are mostly found in peripheral tissues [5]. In the brain, the CB_1_ receptors are abundantly expressed in the hippocampus, cerebellum and striatum [6,7]. Among the peripheral tissues wherein the CB_1_ receptors have been found, the enteric nervous system [8], testis, urinary bladder, vas deferens can be mentioned [9]. CB_1_ receptor mRNA and protein have been furthermore identified in the rat and human eye, both in the retina and in the iris, and in the ciliary body [10]. CB_2_ receptors are located in the marginal zones of the spleen, tonsils, immune cells [9,11] and to a much lesser extent in CNS [12]. Furthermore, CB_2_ receptor mRNA was expressed in the adult rat retina, including the somas of retinal ganglion cells [13].

During the last years, several ligands endowed with high affinities and subtype selectivity for both receptors were identified. Such ligands were proposed as potential therapeutic targets for the treatment of several diseases, including neuropathic pain [14], cancer [15,16] and osteopor4osis [17].

Non-selective CB_1_/CB_2_ receptor agonists are the constituents of some approved medicines, i.e., Sativex^®^, Cesamet^®^, Marinol^®^. Sativex contains approximately equal amounts of Δ^9^-tetrahydrocannabinol and the non-psychoactive phytocannabinoid cannabidiol, and is prescribed for neuropathic pain relief in adults with multiple sclerosis and as an adjunctive analgesic treatment for adult patients in advanced cancer [18]. Furthermore, the relevance of CB receptors as an emerging target of pharmacotherapy is documented also by the discovery of mixed CB_1_/CB_2_ receptor agonists as antiglaucoma agents [19].

**Figure 2:**
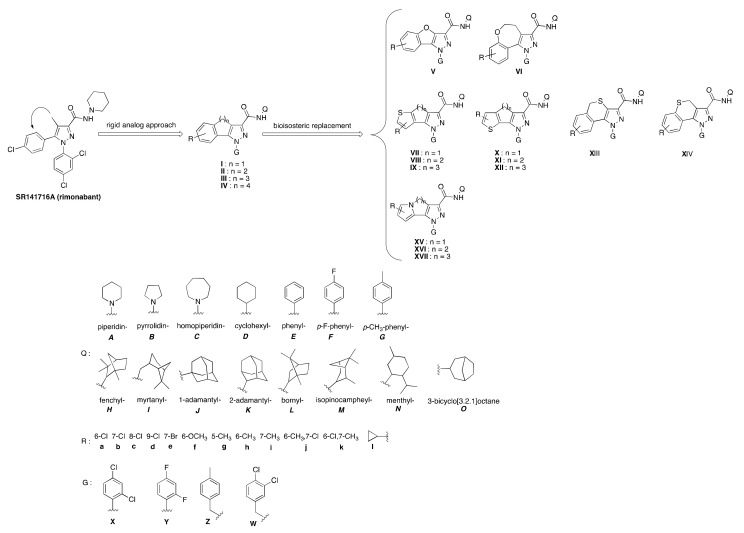
Design of cannabinoid CB_1_/CB_2_ receptor ligands.

The only cannabinoid receptor antagonist approved as a medicine to-date is the CB_1_ antagonist/inverse agonist SR141716A (rimonabant, Acomplia^®^, Figure 1). This compound has been developed for the treatment of obesity and related metabolic risk factors [20]. However, it was soon withdrawn for its serious psychiatric disorders including anxiety, depression and suicidal tendency. Although most pharmaceutical companies were deterred from developing a drug that displayed rimonabant-like CB_1_ receptor antagonist/inverse agonist activity for the management of any disorders, this compound still remains an extremely valuable lead for the design of new ligands for CB receptors interaction [21,22]. Within this frame, the 4-alkyl-5-arylpyrazole skeleton of rimonabant has been modified, leading to benzocycloalkylpyrazole-based tricyclic systems of general formula **I-IV** (Figure 2). Modifications carried out on such scaffolds, allowed to accede to further pyrazole-based tricyclic systems of general formula **V-XVII** (Figure 2). Fine tuning of these tricyclic systems (vide infra) allowed to identify hundreds of molecules, most of which endowed with high affinity and selectivity for CB_1_ or CB_2_ receptors. In this review, pyrazole-based tricyclic compounds **I-XVII** are divided into four main groups according to the size of the central ring connected to the pyrazole one. If a five-membered ring is connected to the pyrazole, the compound belongs to (5,5)-condensed pyrazole derivatives, and if the connected ring is a six-, seven- or eight-membered ring, the compound belongs to (5,6)-, (5,7)- or (5,8)-condensed pyrazole derivatives. Here, we briefly present the synthesis and the bioactivities of pyrazole-based tricyclic compounds **I-XVII** investigated by us and other groups. Furthermore, this review surveys chemical and biological literature of some miscellaneous molecules featuring tricyclic pyrazole structure closely related to compounds **I-XVII**, investigated for cannabinoid receptor affinity.

## 2. (5,5)-Condensed Pyrazole Derivatives

### 2.1. 1,4-dihydroindeno(1,2-c)pyrazole-Based Derivatives

The first (5,5)-condensed pyrazole derivatives synthesized by us for cannabinoid receptors interaction are those of general formula **I** (Figure 2) featuring the 1,4-dihydroindeno(1,2-*c*)pyrazole core. These compounds were designed as rigid analogs of CB_1_ antagonist/inverse agonist SR141716A (rimonabant), endowed with a high affinity for CB_1_ receptors and good selectivity over CB_2_ receptors. It has been proposed that minimization of the flexibility of the lead compound through the introduction of structural constraints could have some impact on cannabinoid receptor interaction, maybe allowing the ligand to bind with high affinity and selectivity to its receptor. Accordingly, the first series of 1,4-dihydroindeno(1,2-*c*)pyrazole derivatives have been synthesized and evaluated for their in vitro binding affinities for CB_1_ and CB_2_ receptors. Most of these compounds are *N*-piperidine-carboxamides that differ for R substituent (Cl, F, I, CH_3_, OCH_3_), whereas **G**, quite often, maintained the 2,4-Cl-phenyl substitution pattern of rimonabant [23].

**Scheme 1:**
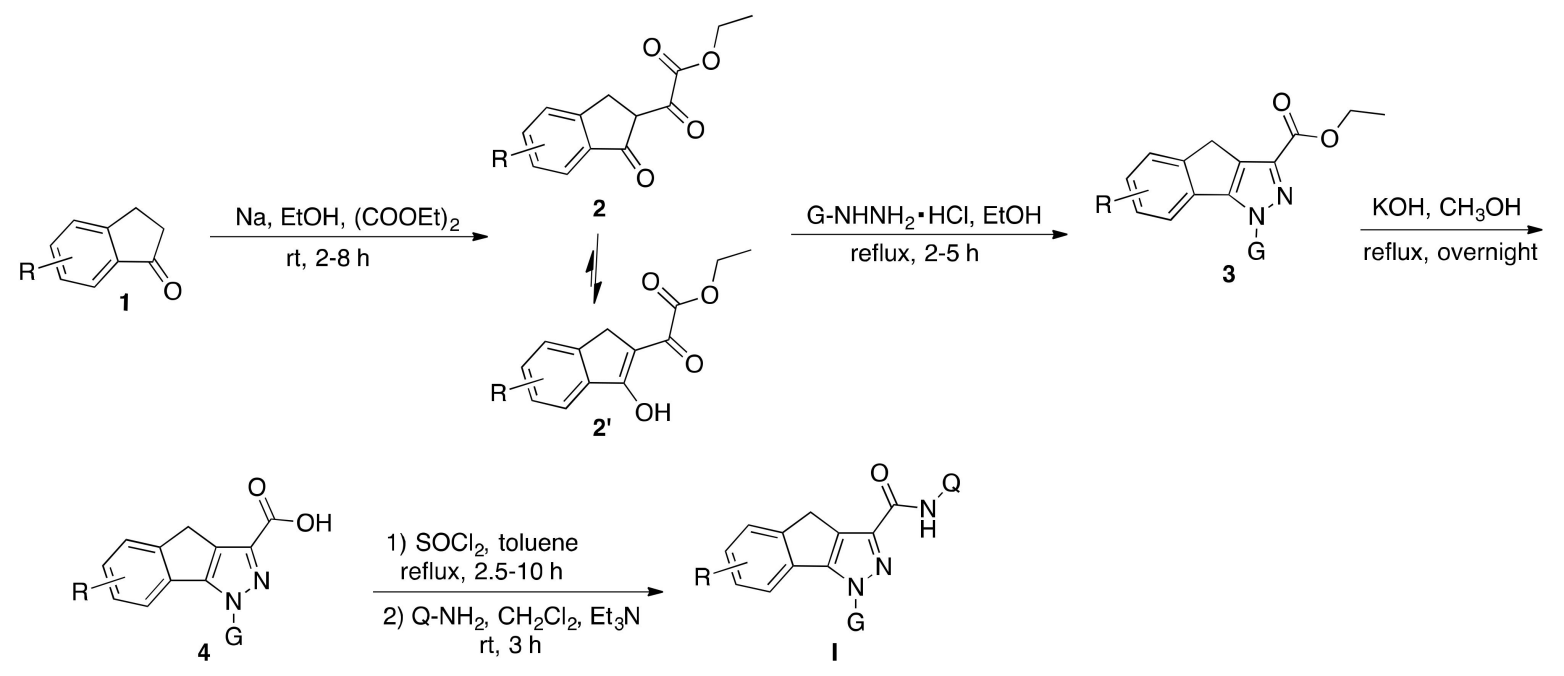
General synthetic route for the preparation of compounds **I**.

Compounds **I** were synthesized starting from the appropriate ketones **1** (Scheme 1) which were α-acylated by Claisen reaction, furnishing 1,3-diketoesters **2** as a tautomeric equilibrium shifted towards the alkenylidene structure (**2′**). The cyclization with appropriate hydrazines in refluxing EtOH gave the tricyclic dihydroindeno(1,2-*c*)pyrazole esters **3**, which were hydrolyzed with KOH, affording the corresponding acids **4**. Target compounds **I** were synthesized by condensation of acids **4**, previously activated to acyl chlorides with SOCl_2_, with the appropriate amines.

If not otherwise stated, the four-step general synthetic route for **I** compounds, with minor changes, was employed for the preparation of all compounds of general formula **II-XVII**, starting from the appropriate ketones and using the appropriate hydrazines and amines.

**Figure 3:**
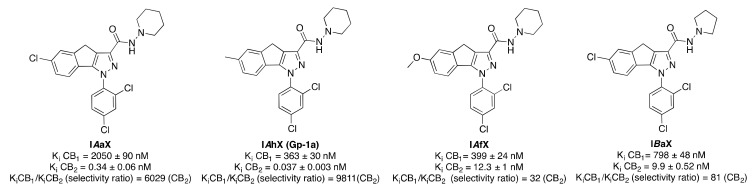
Structure of representative 1,4-dihydroindeno(1,2-*c*)pyrazoles (**I**). Affinities at CB_1_ and CB_2_ receptors were assessed by competition of (^3^H)-CP 55,940 in mouse cerebellum membranes and in mouse spleen homogenates, respectively [23]. Permission has been obtained and there is no copyright issue.

Our first SAR study revealed that several compounds incorporating the planar 1,4-dihydroindeno(1,2-*c*)pyrazole scaffold displayed very high in vitro binding affinity for CB_2_ receptors comparable to, or exceeding, that of SR144528 (Figure 1), identified as the first highly potent and selective ligand for the CB_2_ receptors. Representative derivatives are **I*A*aX**, **I*A*hX**, **I*A*fX**, **I*B*aX** (Figure 3), compound **I*A*hX** emerging for its high affinity for CB_2_ receptors and exceptional selectivity over CB_1_ receptor (CB_1_/CB_2_ selectivity ratio of 9811).

**Figure 4:**
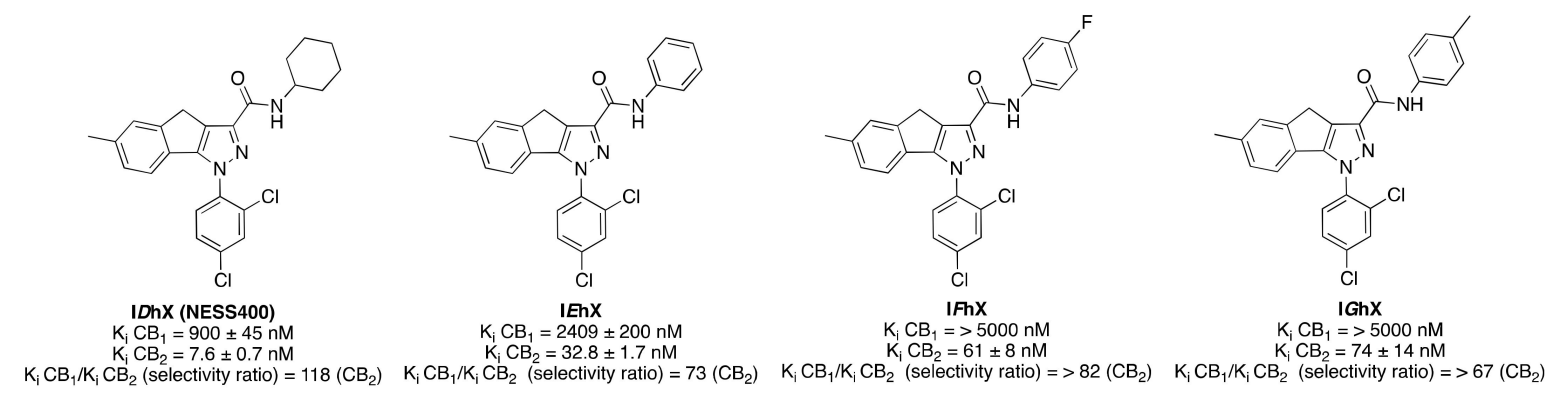
Structure of representative 1,4-dihydroindeno(1,2-*c*)pyrazoles (**I**) obtained by modification of carboxamide moiety of compound **I*A*hX**. Affinities at CB_1_ and CB_2_ receptors were assessed by competition of (^3^H)-CP 55,940 in mouse cerebellum membranes and in mouse spleen homogenates, respectively [24].

Our results prompted us to investigate new 1,4-dihydroindeno(1,2-*c*)pyrazoles (**I**), which were obtained modifying the *N*-carboxamide moiety (**Q**) and the aryl substitution of the lead compounds **I*A*aX** and **I*A*hX** [24]. In general, our SAR study showed that the CB_1_ receptor affinities of all the investigated compounds were lower than their CB_2_ receptor affinities, evidencing the suitability of 1,4-dihydroindeno(1,2-*c*)pyrazole architecture to generate ligands for CB_2_ receptor interaction. Representative compounds of this series are **I*D*hX**, **I*E*hX**, **I*F*hX**, **I*G*hX** (Figure 4), all displaying high CB_2_ affinity and reasonable selectivity over CB_1_ receptors, even if lower than that elicited by compounds **I*A*aX** and **I*A*hX**.

In vitro CB_2_ intrinsic activity evaluation assay, based on the determination of P-ERK 1/2 increasing expression in human promyelocitic leukemia HL-60 cells exposed to the compounds to be assayed, highlighted agonist activity toward CB_2_ receptors for derivatives **I*D*hX**, **I*E*hX** and for prototype **I*A*aX**, **I*A*hX**. In particular, the tested compounds significantly increased P-ERK 1/2 expression, reaching the maximum effect at the concentration of 10 nM with the following values: +61.3 ± 12.4% (**I*D*hX**), +125.0 ± 35.8% (**I*E*hX**), +65.0 ± 18.5% (**I*A*aX**), +83.0 ± 4.2% (**I*A*hX**) versus vehicle. Overall, the effect of tested compounds as CB_2_ agonists was specific as it was blocked by the CB_2_ receptor antagonist SR144528 [24].

**Figure 5:**
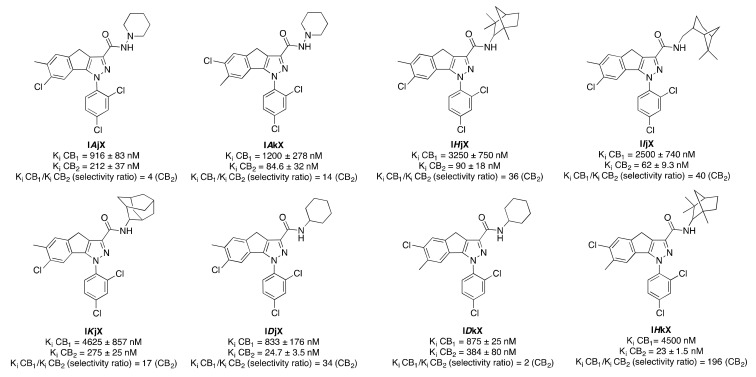
Structure of representative 1,4-dihydroindeno(1,2-*c*)pyrazoles (**I**) designed making use of molecular hybridization based on scaffold hopping. Affinities at CB_1_ and CB_2_ receptors were assessed by competition of (^3^H)-CP 55,940 in mouse cerebellum membranes and in mouse spleen homogenates, respectively [26].

By pursuing our interest in expanding SAR studies around 1,4-dihydroindeno(1,2-*c*)pyrazole core, especially in the light of the potential of CB_2_ ligands for the treatment of immune disorders or as anti-nociceptive agents [14,25], novel derivatives have been designed, making use of molecular hybridization based on scaffold hopping [26]. In particular, based upon the putative interacting sites and structural features of selective CB_2_ antagonist SR144528 (Figure 1), i.e., *N*_1_-benzyl group, the C_3_ carboxamide moiety, and the substitution of the C_5_ phenyl ring [26,27], it was postulated that the introduction of such pharmacophoric elements in the tricyclic core of compound **I*A*hX** might provide new CB_2_ ligands with potential therapeutic value. Briefly, different synthesized compounds were monoterpene derivatives incorporating bulky groups in the carboxamide moiety (i.e., fenchyl-, bornyl-, isopinocampheyl-, myrtanyl-, menthyl-), bearing a 6-CH_3_,7-Cl or 6-Cl,7-CH_3_ substitution pattern at the aryl ring of 1,4-dihydroindeno(1,2-*c*)pyrazole core. Some adamantane derivatives were also investigated, together with compounds incorporating simple cycloalkyl motifs at the carboxamide moiety. Compounds **I*A*jX, I*A*kX, I*H*jX, I*I*jX, I*K*jX, I*D*jX, I*D*kX, I*H*kX** (Figure 5) are representatives of these third series of **I** compounds. As described above for representative compounds depicted in Figure 3 and Figure 4, our SAR study carried out on a third series of molecules, evidenced for 1,4-dihydroindeno(1,2-*c*)pyrazole-based molecules preferential affinity for CB_2_ receptors. However, the introduction of a chlorine atom, as well as its exchange with the methyl group in all-new hybrid compounds seemed to play a modest role in lowering the levels of CB_2_-affinity as compared to the reference compounds **I*A*aX** and **I*A*hX**.

According to previous data obtained for compounds **I*A*aX**, **I*A*hX**, **I*D*hX** and **I*E*hX**, analogs **I*I*jX, I*D*jX** and **I*H*kX**, endowed with the highest affinity for CB_2_ receptors, exhibited CB_2_ agonism activity in in vitro model based on the determination of P-ERK 1/2 increasing expression in HL-60 cells, with maximum values reached at 125 nM for compounds **I*I*jX** (+54.5 ± 12.1%) and **I*D*jX** (+82.5 ± 19.1%), and at 50 nM for compound **I*H*kX** (+46.4 ± 15.6%) versus vehicle. Furthermore, the effect of such tested compounds on P-ERK ½ expression was counteracted by the reference CB_2_ antagonist AM630, suggesting the correspondence between the detected effect and CB_2_ modulation.

The obtained data confirmed that the flattening of 1,4-dihydroindeno(1,2-*c*)pyrazole core is important to assure agonist rather than antagonist activity, with respect to not condensed and more flexible analogs such as the antagonist SR144528.

Within this frame, a series of compounds incorporating the 1,4-dihydroindeno(1,2-*c*)pyrazole scaffold (**I**), sharing the *N*_1_-benzyl group and bulky groups in the carboxamide moiety (i.e., **I*O*kW**, Figure 2) were claimed by Sanofi-Aventis, but no specific biological activity was presented [28]. However, the compounds of the invention were generally described as potent and selective CB_2_ receptor antagonists with Ki values < 5 × 10^−7^ M. Antagonistic properties of such compounds have been demonstrated by the results in the models for inhibition of adenylate cyclase induced by forskolin, although no specific examples or data were disclosed.

**Figure 6:**
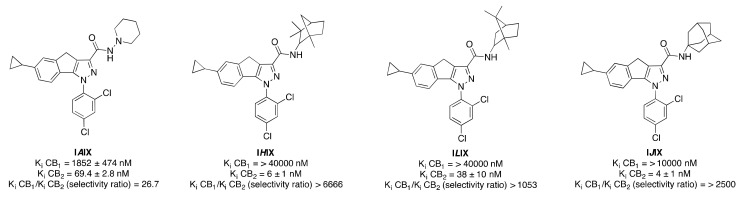
Structure and CB_1_/CB_2_ binding affinities of representative cyclopropyl-based 1,4-dihydroindeno(1,2-*c*)pyrazoles (**I**). Affinities at CB_1_ and CB_2_ receptors were evaluated by competition of (^3^H)-CP 55,940 in human CB_1_ or CB_2_ receptors transfected into HEK293 EBNA cells [29].

Furthermore, 1,4-dihydroindeno(1,2-*c*)pyrazoles featuring a cyclopropyl or cyclohexyl building block in C_6_ position were investigated by us, in order to evaluate the effect of cycloalkyl moiety in place of methyl group both on cannabinoid receptor binding and activity [29]. The most interesting compounds coming from this fourth series are depicted in Figure 6. Whereas, these analogs provided further insight regarding the structural features for CB_2_ affinity and selectivity, our data evidenced that the introduction of a cyclopropyl moiety in most of the synthesized compounds seemed to play a modest role in lowering the CB_2_ receptor affinity, especially if compared to compound **I*A*hX**. Interestingly, intrinsic activity for compounds **I*A*lX, I*H*lX, I*L*lX, I*J*lX**, evaluated by GTPγS binding assay, showed antagonist/inverse agonist properties (IC_50_ for compound **I*A*lX** = 294 nM, for **I*H*lX** = 80 nM, for **I*L*lX** = 27 nM and for **I*J*lX** = 51 nM).

Independently, other groups investigated the biological properties of some 1,4-dihydroindeno(1,2-*c*])pyrazoles synthesized by our group. In particular, within the search aiming to characterize the molecular pharmacology of the most widely used CB_2_ receptor ligands, it was reported for compound **I*A*hX**, namely Gp-1a, an affinity for the CB_2_ receptors markedly different from that reported by our group (K_i_ CB_1_ = 426 ± 0.08 nM, K_i_ CB_2_ = 20.9 ± 0.23 nM; CB_1_/CB_2_ selectivity ratio = 20), using (^3^H)CP-55,940 and mouse brain and spleen as source for CB_1_ and CB_2_ receptors, respectively [30]. Furthermore, in contrast to our results, the same authors reported for Gp-1a inverse agonist properties on CB_2_ receptors in GTPγS assay and resulted not active in hCB_2_ receptor pERK assay [30]. Indeed, the first two series of 1,4-dihydroindeno(1,2-*c*)pyrazole-based compounds synthesized by our group [23,24], including Gp-1a, were described in a patent application providing methods and pharmaceutical compositions for reducing the serum level of immunoglobulin IgE in an animal or human subject [31]. In particular, it was reported the CB_2_ agonist Gp-1a attenuated the serum levels of total IgE from BALB/e mice and the co-treatment with the CB_2_ antagonist SR144528 reversed the Gp-1a effect. Therefore, the patent provided a method for modulating this type of antibody for the treatment of immune system-related conditions such as allergy, hay fever and the like. Biological literature concerning Gp-1a pointed out the relevance of such a compound as a useful pharmacological tool to ascertain in more detail the role of CB_2_ receptors in physiopathological conditions [32,33,34,35,36]. Furthermore, the pharmacological relevance of 1,4-dihydroindeno(1,2-*c*)pyrazole-based **I** compounds emerged also from the significant anti-nociceptive activity of the potent and selective CB_2_ agonist **I*D*hX**, namely NESS400, in Spared Nerve Injury (SNI) neuropathic mice, by alleviating both mechanical allodynia and thermal hyperalgesia [37].

### 2.2. Benzofuro(3,2-c)pyrazol-Based Derivatives (**V**)

**Figure 7:**
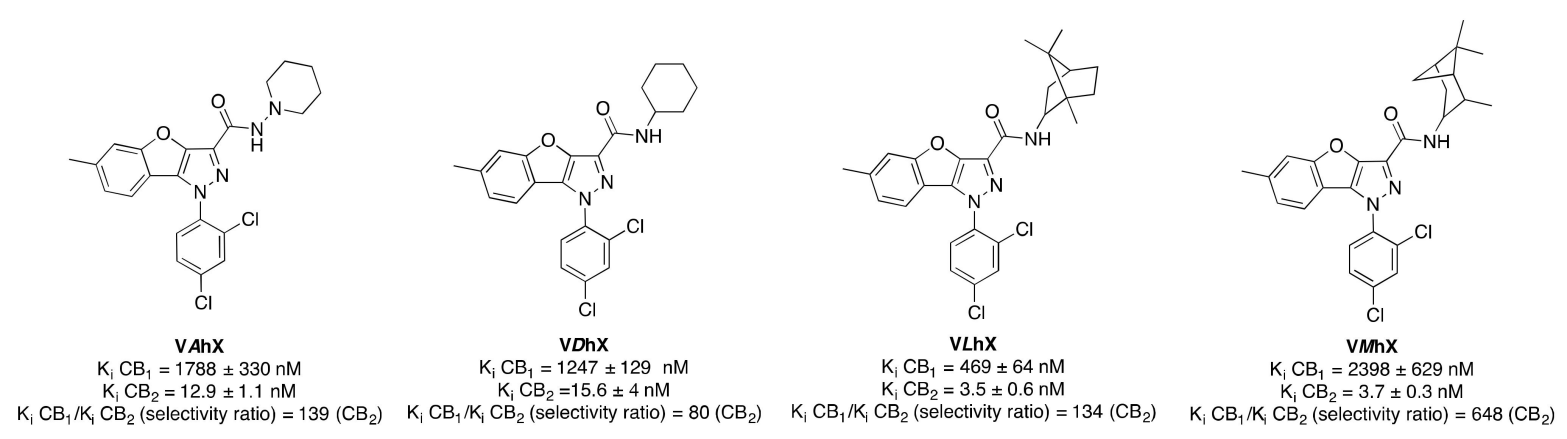
Structure of representative benzofuro(3,2-*c*)pyrazol-based derivatives (**V**). Affinities at CB_1_ and CB_2_ receptors were assessed by competition of (^3^H)-CP 55,940 in mouse cerebellum membranes and in mouse spleen homogenates, respectively [38].

Further condensed (5,5)-pyrazole derivatives were designed by us making use of bioisosteric replacement as drug design approach to obtain novel planar tricyclic scaffold for cannabinoid receptor interaction [38]. Following this approach, a new series of 1,4-dihydroindeno(1,2-*c*)pyrazole analogs, namely benzofuro(3,2-*c*)pyrazoles (**V**, Figure 2), containing an oxygen atom at position 4 instead of a methylene unit, were designed. The structure of representative compounds is depicted in Figure 7. These compounds were previously described by Neuroscienze Pharmaness S.C.A.R.L in a patent application claiming specific composition of microemulsions of pharmaceutical compounds. Indeed, the patent covers a more extensive series of tricyclic pyrazoles belonging both to (5,5) as well as (5,6) and (5,7]) series [39].

In general, our SAR study revealed high CB_2_ receptor affinity for the new compounds, with values qualitatively similar with those of compound **I*D*hX**, as well as those of related analogs incorporating the 1,4-dihydroindeno(1,2-*c*)pyrazole skeleton. In contrast, a comparison of K_i_CB_2_ value of compound **I*A*hX** and that of the analog **V*A*hX** revealed two orders of magnitude decreased affinity for **V*A*hX**. Compounds **V*L*hX** and **V*M*hX**, featuring a bornyl and isopinocampheyl moiety at the carboxamide portion, exhibited the best CB_2_ cannabinoid binding profiles among all synthesized derivatives. CB_2_ functional assay carried out on HL-60 cells, based on the evaluation of P-ERK1/2 expression induced by cannabinoid ligands, evidenced agonism behavior for all synthesized compounds [38], with calculated maximum values, in most cases, exceeding that of the agonist WIN-55,212-2 (*i.e*., **V*A*hX**: dose 50 nM, 205 ± 12%; **V*D*hX**, dose 50 nM, 196 ± 20%; **V*L*hX**: dose 10 nM, 189 ± 11%; **V*M*hX**: dose 5 nM, 205 ± 6%; WIN-55,212-2: dose 75 nM, 190 ± 17% versus vehicle). Overall, the effect of tested compounds as CB_2_ agonists was specific as it was blocked by the CB_2_ receptor antagonist AM630 [38].

### 2.3. 1,4-Dihydrothieno(2′,3′-4,5)cyclopenta(1,2-c)pyrazole-Based Derivatives (**VII**) and 1,4-Dihydrothieno(3′,2′-4,5)cyclopenta(1,2-c])pyrazole-Based Derivatives (**X**)

**Figure 8:**
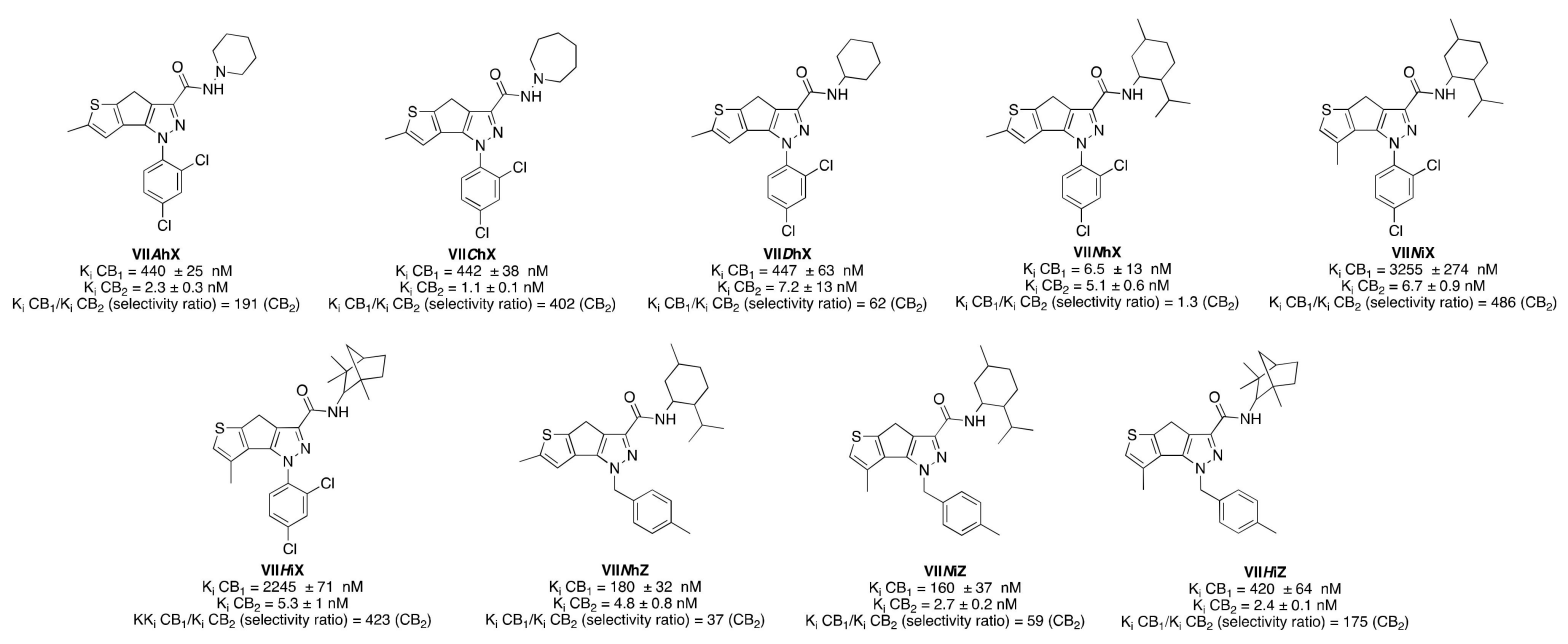
Structure of representative 1,4-dihydrothieno(2′,3′-4,5)cyclopenta(1,2-*c*)pyrazole-based derivatives (**VII**). Affinities at CB_1_ and CB_2_ receptors were assessed by competition of (^3^H)-CP 55,940 in mouse cerebellum membranes and in mouse spleen homogenates, respectively [40].

To further investigate the versatility of (5,5)-pyrazole condensed tricyclic derivatives in the development of CB_2_ ligands as therapeutic agents, our attention was focused on the benzene ring of the tricyclic indenopyrazole scaffold (**I**), by replacing it with a thiophene ring, giving rise the novel dihydrothienocyclopentapyrazole architecture which fine-tuning furnished novel derivatives with general formula **VII** (Figure 2). It was postulated that bioisosteric replacement benzene/thiophene could be an efficient strategy to develop novel CB_2_ selective ligands and maybe provide further insight concerning structural features for cannabinoid receptor interaction [40]. In our SAR study, we planned to investigate the effect of changing the size of carboxamide moiety (**Q**), of methyl shifting from C_6_ to C_7_ of the tricyclic platform, as well as the effect related to the replacement of the *N*_1_-dichlorophenyl group (**X**) with *p*-methylbenzyl moiety (**Z**) on cannabinoid receptor affinity. The structure of representative compounds is depicted in Figure 8. The major term, compound **VII*A*hX**, displayed a high affinity for CB_2_ receptors, even if 62-fold decreased with respect to the reference compound **I*A*hX** with selectivity ratio K_i_ CB_1_/K_i_ CB_2_ = 191. A similar trend was observed in several thienocyclopentapyrazole compounds (Figure 8) with the exception of **VII*N*hX**, which exhibited a mixed binding profile, reaching K_i_ values of 6.5 and 5.1 nM for CB_1_ and CB_2_, respectively. All these compounds profiled as full agonists at CB_2_ receptors in an assay based on the determination of P-ERK 1/2 increasing expression in HL-60 cells [40].

**Figure 9:**
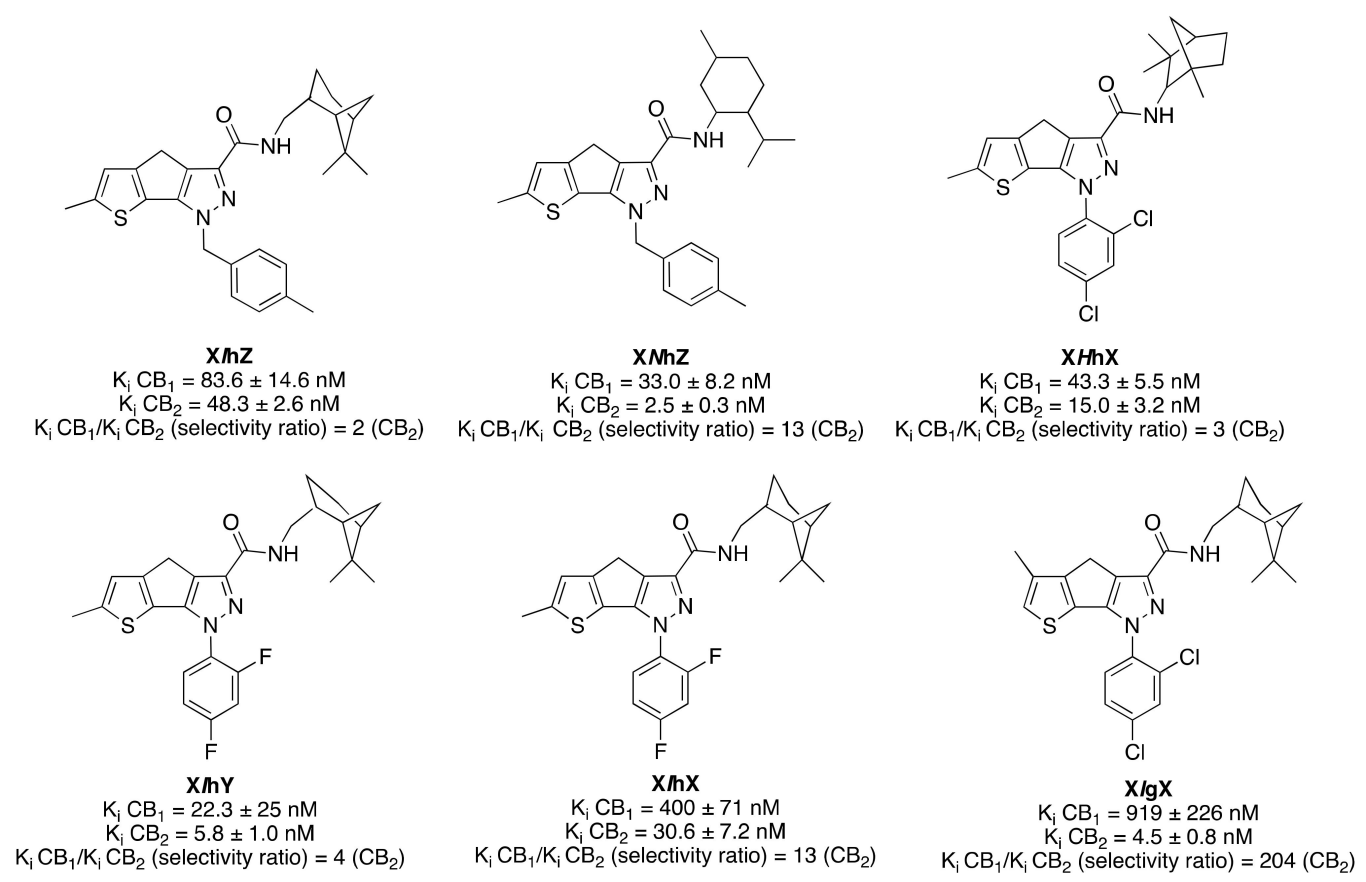
Structure of representative 1,4-dihydrothieno(3′,2′-4,5)cyclopenta(1,2-*c*)pyrazole-based derivatives (**X**). Affinities at CB_1_ and CB_2_ receptors were assessed by competition of (^3^H)-CP 55,940 in mouse cerebellum membranes and in mouse spleen homogenates, respectively [41].

Within this framework, a series of 1,4-dihydrothieno(3′,2′-4,5)cyclopenta(1,2-*c*)pyrazole-based derivatives with general formula **X** (Figure 2) were investigated. Representative compounds are depicted in Figure 9. Such compounds were described by Neuroscienze Pharmaness S.C.A.R.L in a patent application encompassing a more extensive series of condensed tricyclic pyrazoles [41]. According to binding data, shifting the sulfur atom of the 1,4-dihydrothieno(2′,3′-4,5)cyclopenta(1,2-*c*)pyrazole structural template from position 5 to 7 induced a significant impact on cannabinoid receptor affinity. In general, most of the investigated compounds exhibited a preferential affinity for CB_2_ receptors, with different compounds showing mixed and high CB_1_/CB_2_ cannabinoid receptor affinities (**X*I*hZ, X*N*hZ, X*H*hX, X*I*hY, X*I*hX, X*I*gX**, Figure 9).

Amongst the compounds claimed in the patent application, derivatives **X*I*hZ, X*N*hZ, X*H*hX** were investigated to evaluate their intrinsic activity as agonist or antagonist on CB_1_ receptors in an ex-vivo model based on the use of the vas deferens. According to the behavior of WIN-55,212-2 and other CB_1_ ligands in mice vas deferens isolated organ assay [42], compounds **X*I*hZ, X*N*hZ, X*H*hX** showed agonist activity, with **X*I*hZ** the most effective in inhibiting the contractions induced by an electric stimulus compared to the basal value, as evidenced by the reported dose-response curve [41].

Compounds **X*I*hZ, X*N*hZ, X*H*hX** were also investigated for their ability to reduce intraocular pressure (IOP) which is considered a prominent risk factor for glaucoma development and progression [43]. The first study highlighting the relevance of CB_1_/CB_2_ agonists for the treatment of glaucoma was conducted in 1971, reporting that smoking marijuana significantly lowered the IOP [44]. Very positive results were reported also for WIN-55,212-2, which is the prototype of the aminoalkylindole class of synthetic cannabinoids, activating both CB_1_ and CB_2_ receptors, albeit with a proclivity for the CB_2_ receptors. In particular, in normotensive rabbits, a single dose of WIN-55,212-2, either topical or systemic, significantly reduced IOP without apparent ocular toxicity, most likely through effects on CB_1_ receptors [45]. Subsequent studies showed a reduction of IOP in glaucomatous rats after local and chronic administration of WIN-55,212-2, without adverse effects [46]. These findings are consistent with another experiment showing that WIN-55,212-2 decreased IOP in human glaucoma resistant to conventional therapies [47]. Further studies have demonstrated increased aqueous outflow after exposure to the CB_2_ agonist JWH015, suggesting a beneficial function derived from CB_2_ receptor activation in the treatment of ocular diseases such as glaucoma [48]. Neuroprotective properties of cannabinoids have been demonstrated in CNS neurodegenerative diseases with different mechanisms [49]. Several studies have shown in the retina that CB_1_ agonists (Δ^9^-tetrahydrocannabinol and cannabidiol) protected ganglion cells from glutamate-excitotoxicity and ischemia caused by increased IOP [50,51]. Although all these data are promising, an important issue for the clinical potential of cannabinoids as anti-glaucoma agents has been cardiovascular and psychotropic side effects mediated by systemic and brain cannabinoid receptor activation [52,53,54]. Additionally, short duration of action of cannabinoids on IOP reduction (i.e., the duration of action after smoking marijuana is only 3–4 h) is another issue that has to be overcome in the application of these compounds in treating glaucoma. Actually, standard therapeutic options in treating glaucoma include a few IOP-lowering drugs as prostaglandin analogs, β-adrenoreceptor antagonists, α_2_-adrenoreceptor agonists, carbonic anhydrase inhibitors, and cholinergic agonists [55]. When medical therapy failed to lower IOP, laser or surgical interventions are extremely considered in order to prevent disease progression toward blindness.

According to unmet medical need, compounds **X*I*hZ, X*N*hZ, X*H*hX**, exhibiting high and mixed CB_1_/CB_2_ receptor affinities, with a proclivity for CB_2_ subtype, and endowed of CB_1_ agonist properties, according to the CB receptor profile of the reference cannabinoidergic compound WIN-55,212-2, by using the animal model of old DBA/2J mice, were investigated for their ability to reduce IOP [41]. Compounds **X*I*hZ, X*N*hZ, X*H*hX** and WIN-55,212-2, which was used as reference compound, were dispersed in the commercial emulsion Tocrisolve™ and applied to the eye of old DBA/2J mice at a concentration of 100 µg or 50 µg. The obtained results, reported in Table 1, showed that commercial emulsion Tocrisolve™ (20, 40 µL) had no effect on the IOP. Furthermore, at the dose of 100 µg, all tested compounds were effective in reducing eye pressure as the reference compound WIN-55,212-2, while only **X*I*hZ**, at the dose of 50 µg was more effective in reducing IOP than the reference compound. What is claimed in the patent application was pharmaceutical compositions comprising emulsions or microemulsions may be useful to avoid systemic side effects of such cannabinoid compounds. The preliminary results reported in the patent application [41] for compound **X*I*hZ** could pave the way for the development of novel cannabinoidergic compounds as anti-glaucoma agents.

### 2.4. 1,4-Dihydropyrazolo(3,4-a)pyrrolizine-Based Derivatives (**XV**)

**Figure 10:**
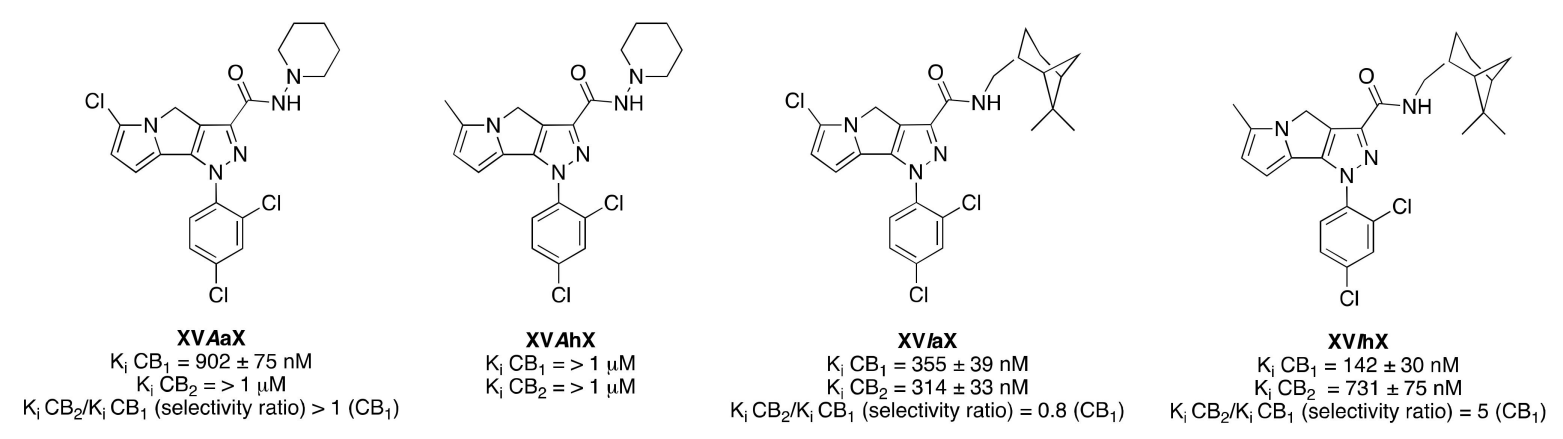
Structure of representative 1,4-dihydropyrazolo(3,4-*a*)pyrrolizine-based derivatives (**XV**). Affinities at CB_1_ and CB_2_ receptors were assessed by competition of (^3^H)-CP 55,940 in mouse whole-brain membranes and in CHO cell membranes transfected with hCB_2_ receptors [56].

Continuing with our interest in expanding SAR studies on cannabinoid receptors, and taking into consideration the binding profile of compounds **I*A*aX** and **I*A*hX**, a new tricyclic pyrazole scaffold, namely 1,4-dihydropyrazolo(3,4-*a*)pyrrolizine **XV**, was designed on the basis of bioisosteric replacement approach (benzene/pyrrole) [56]. Representative compounds are depicted in Figure 10. Surprisingly, none of the new compounds exhibited a high affinity for CB_2_ receptors, with K_i_ values above 314 nM. Negligible affinity was also determined for CB_1_ receptors, with **XV*I*hX** reaching the K_i_ value of 142 nM.

## 3. (5,6)-Condensed Pyrazole Derivatives

### 3.1. 4,5-Dihydro-1H-benzo(g)indazole-Based Derivatives (**II**)

**Figure 11:**
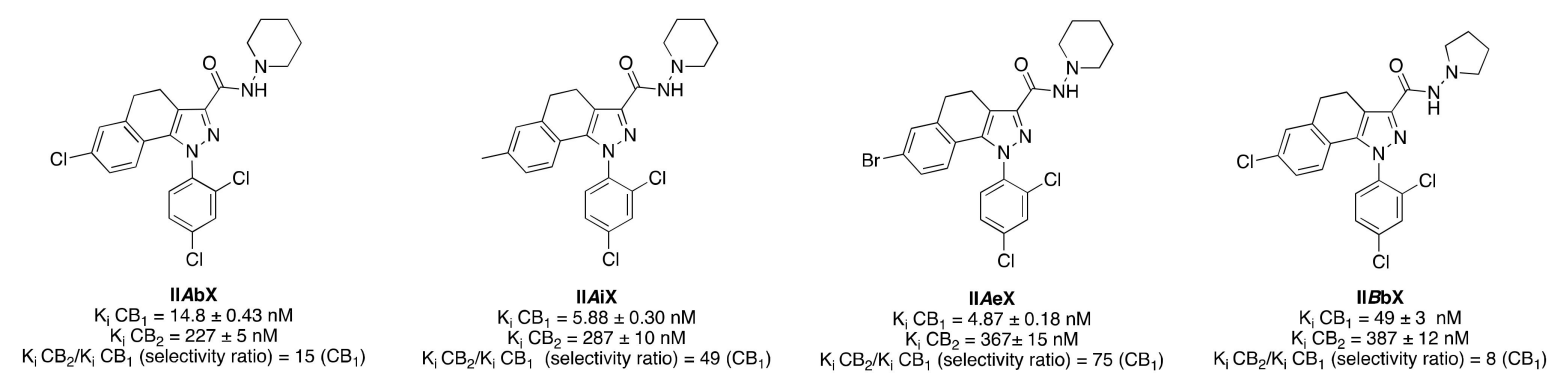
Structure of representative 4,5-dihydro-1*H*-benzo(*g*)indazole-based derivatives (**II**). Affinities at CB_1_ and CB_2_ receptors were assessed by competition of (^3^H)-CP 55,940 in mouse cerebellum membranes and in mouse spleen homogenates, respectively [57].

The first (5,6)-condensed pyrazole derivatives synthesized by us are those of general formula **II** (Figure 2), featuring the 4,5-dihydro-1*H*-benzo(*g*)indazole scaffold which is a homologue of 1,4-dihydroindeno(1,2-*c*)pyrazole one (compounds **I**). Representative compounds are depicted in Figure 11 [57]. Most of such compounds were described by Sanofi-Aventis in a patent application as cannabinoid CB_1_ receptor antagonists, but no biological data were reported [58].

Interestingly, as evidenced by K_i_ values of compounds **II*A*bX** and **II*A*iX** with respect to compounds **I*A*aX** and **I*A*hX**, increasing the carbocyclic central ring size by one methylene unit (from (5,5)- to (5,6)-condensed pyrazole derivatives) involved a marked loss of affinity for CB_2_ receptors, a significant increase in CB_1_ affinity, and a consequent loss of CB_2_ selectivity. A similar trend was exhibited by other derivatives incorporating such 4,5-dihydro-1*H*-benzo(*g*)indazole scaffold (i.e., **II*A*eX** and **II*B*bX**). The SAR study carried out on the two homologous series **I** and **II**, prompted us to suppose that to achieve high binding affinity to CB_1_ receptors and CB_1_ over CB_2_ selectivity it is important for the tricyclic system to be non-planar. To evaluate the functional profile, several compounds were assayed for the capability to affect gastrointestinal transit in mice, making use of the upper gastrointestinal test which is based on the determination of the intestinal length traveled by a non-absorbable marker as a consequence of the active compound administration. From this test, compound **II*A*bX** was able to induce a dose-dependent gastrointestinal motility increase, as well as compound **II*A*iX**. This effect was markedly reversed by the in vivo administration of CP-55,940, suggesting for the series of 4,5-dihydro-1*H*-benzo(*g*)indazole-3-carboxamides antagonistic profile for CB receptors [57].

### 3.2. 4,5-Dihydro-1H-thieno(2,3-g)indazole-Based Derivatives (**VIII**) and 4,5-Dihydro-1H-thieno(3,2-g)indazole-Based Derivatives (**XI**)

**Figure 12:**
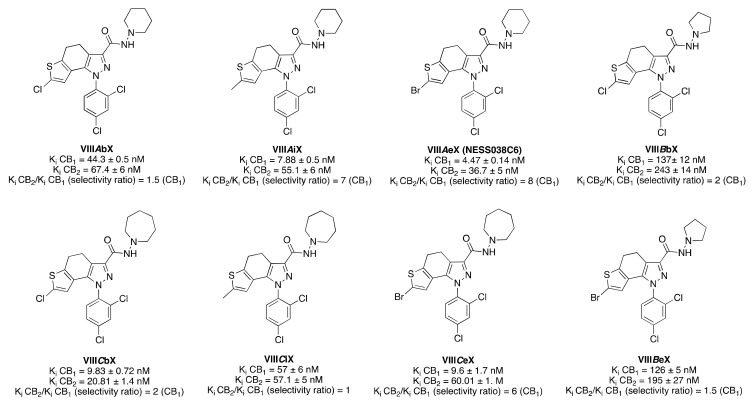
Structure of representative 4,5-dihydro-1*H*-thieno(2,3-*g*)indazole-based derivatives (**VIII**). Affinities at CB_1_ and CB_2_ receptors were assessed by competition of (^3^H)-CP 55,940 in mouse cerebellum membranes and in mouse spleen homogenates, respectively [59].

A number of compounds sharing a 4,5-dihydro-1*H*-thieno(2,3-*g*)indazole scaffold (**VIII**), which is a homolog of the previously-described 1,4-dihydrothieno(2′,3′-4,5)cyclopenta(1,2-*c*)pyrazole one (**VII**) were claimed by Neuroscienze Pharmaness S.C.A.R.L. [59]. The structure of representative compounds and CB_1_/CB_2_ receptor affinity values are reported in Figure 12. According to binding data, most compounds exhibited a potent and mixed CB_1_/CB_2_ binding profile, with a proclivity for CB_1_ receptors. The thieno(2,3-*g*)indazole-based derivative **VIII*A*eX** resulted in the most selective for CB_1_ receptors between all reported compounds. The disclosed compound **VIII*A*eX**, namely NESS038C6, highlighted CB_1_ antagonism behavior by both in isolated organ assays and in vivo tests based on rat intestinal motility (data not shown). Chronic treatment with NESS038C6 in C57BL/6N diet-induced obesity (DIO) mice determined a significant reduction of weight which was comparable to that detected in DIO mice treated with SR141716 [60]. The Neuroscienze Pharmaness S.C.A.R.L. patent [59] encompasses also a series of isomeric compounds incorporating the 4,5-dihydro-1*H*-thieno(3,2-*g*)indazole scaffold **XI** (i.e., **XI*A*bX**, Figure 2) and others, but not cannabinoid binding receptor affinity was reported.

### 3.3. 1,5-Dihydroisothiochromen([4,3-c)pyrazole-Based Derivatives (**XIII**) and 1,4-Dihydrothiochromeno(4,3-c)pyrazole-Based Derivatives (**XIV**)

A series of derivatives featuring a 1,5-dihydroisothiochromeno(4,3-*c*]pyrazole- and 1,4-dihydrothiochromeno(4,3-*c*)pyrazole-scaffolds, **XIII** and **XIV**, respectively (i.e., **XIII*B*bX** or **XIV*A*bX**, Figure 2) were claimed by Sanofi-Aventis for cannabinoid receptor interaction, but no biological activity was presented [58].

### 3.4. 4,5-Dihydro-1H-Pyrazolo(4,3-g)indolizine-Based Derivatives (**XVI**)

**Figure 13:**
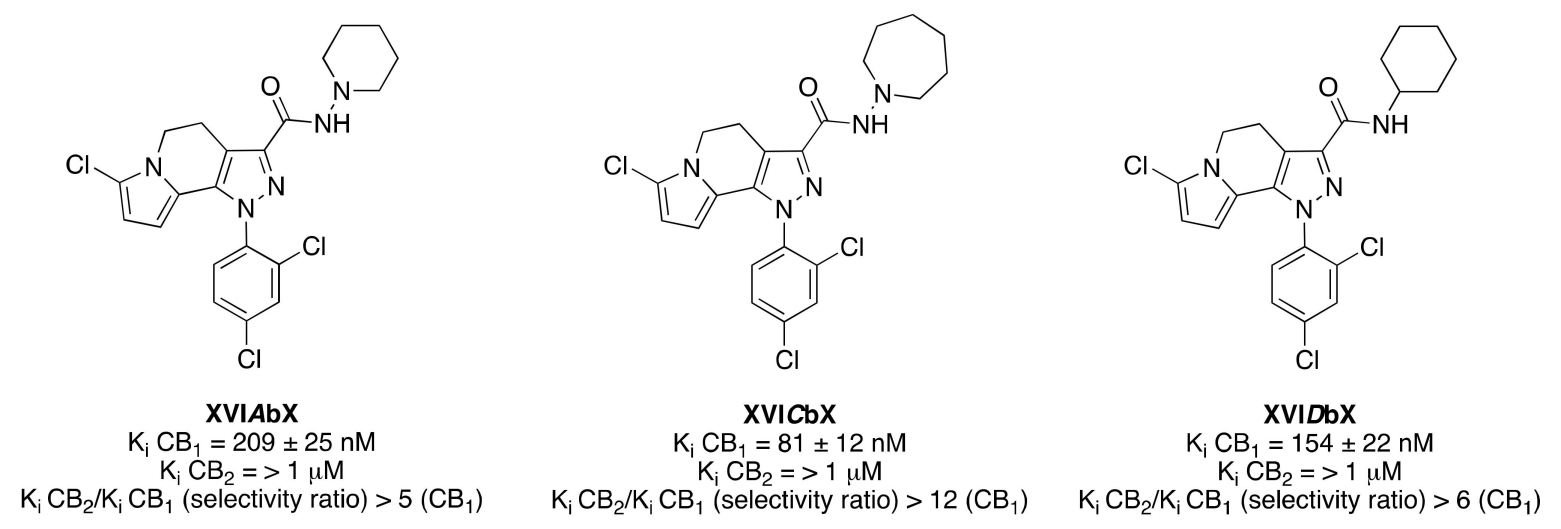
Structure of representative 4,5-dihydro-1*H*-pyrazolo(4,3-*g*)indolizine-based derivatives (**XVI**). Affinities at CB_1_ and CB_2_ receptors were assessed by competition of (^3^H)-CP 55,940 in mouse whole-brain membranes and in CHO cell membranes transfected with hCB_2_ receptors [56].

To further extend SAR study on cannabinoid receptors, and driven by the negligible results on CB_1_/CB_2_ receptor affinity of 1,4-dihydropyrazolo(3,4-*a*)pyrrolizines (**XV**), homologue 4,5-dihydro-1*H*-pyrazolo(4,3-*g*)indolizines (**XVI, Figure 2**) have been synthesized [56]. Representative compounds are depicted in Figure 13. According to binding data reported for compounds **XVI*A*bX**, **XVI*C*bX, XVI*D*bX** with respect to compounds **XV*A*aX, XV*A*hX, XV*I*aX, XV*I*hX**, the expansion of the central ring size by a methylene unit led to conformational changes that promote the affinity for CB_1_ receptors and improve CB_2_/CB_1_ selectivity ratio, with **XVI*C*bX** reaching reasonable affinity for CB_1_ receptors (K_i_ CB_1_ = 81 nM) and the highest CB_2_/CB_1_ selectivity ratio (>12).

## 4. (5,7)-Condensed Pyrazole Derivatives

### 4.1. 1,4,5,6-Tetrahydrobenzo(6,7])cyclohepta(1,2-c]pyrazole-Based Derivatives (**III**)

**Figure 14:**
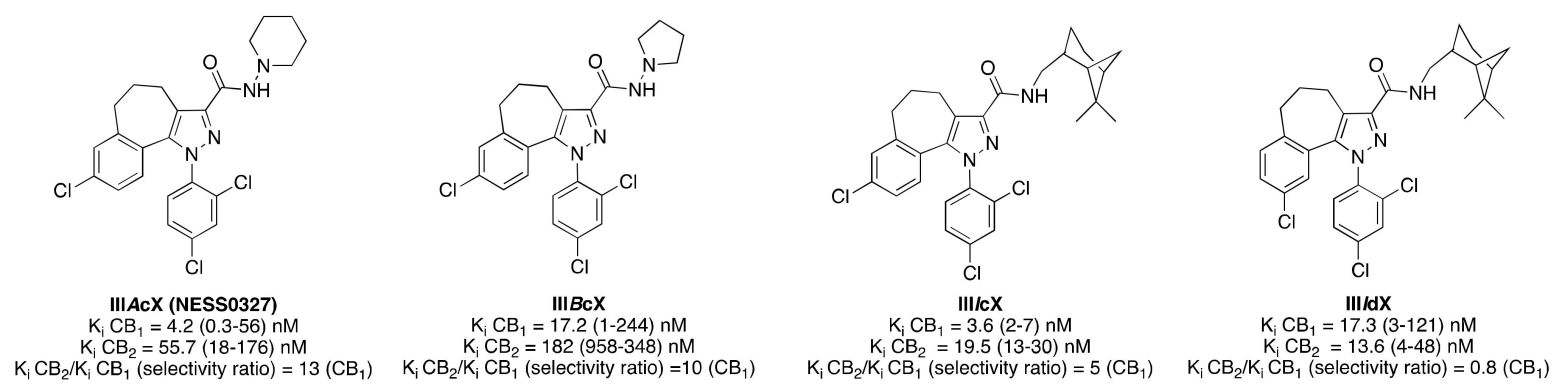
Structure of representative 1,4,5,6-tetrahydrobenzo(6,7)cyclohepta(1,2-*c*)pyrazole-based derivatives (**III**). Affinities at CB_1_ and CB_2_ receptors were assessed by competition of (^3^H)-CP 55,940 in mouse whole-brain membranes and in CHO cell membranes transfected with hCB_2_ receptors [61].

The intriguing SAR study carried out on ligands with the 1,4-dihydroindeno(1,2-*c*)pyrazole core (i.e., **I*A*aX, I*A*hX**) endowed with very high binding affinity for CB_2_ receptors in comparison to homologous ligands having 4,5-dihydro-1*H*-benzo(*g*)indazoles (i.e., **II*A*bX, II*A*iX**) exhibiting higher CB_1_ binding affinity, prompted us to design and synthetize a new homologous series of general formula **III**, incorporating 1,4,5,6-tetrahydrobenzo(6,7)cyclohepta(1,2-*c*)pyrazole scaffold, for cannabinoid receptor interaction [61]. Representative compounds are depicted in Figure 14. Compound **III*A*cX**, namely NESS0327, was first claimed by Sanofi-Aventis, but no biological data was presented in the patent [58].

In general, all the investigated compounds exhibited preferential affinity for CB_1_ receptors, with NESS0327, endowed of K_i_ CB_1_ = 4.2 nM, K_i_ CB_2_ = 55.7 nM and K_i_ CB_2_/K_i_ CB_1_ selectivity ratio = 13.26. Slightly different values for CB_1_ receptors, possibly due to different receptor matrix, were reported for **III*A*cX** by two other independent groups: Stoit et al. K_i_ CB_1_ = 126 nM [62] and Zhang et al. 18.4 nM [63] using (^3^H)-CP 55,940 and hCB_1_ receptor cloned in CHO cells or membranes isolated from a HEK-293 expression system, respectively. Compound **III*I*cX**, bearing the bulky myrtanil substituent, showed the best CB_1_ receptor affinity which was equivalent to that exhibited by **III*A*cX**. Moving the chlorine atom from position 8 to 9 of the myrtanil-based derivative **III*I*cX** to give **III*I*dX** induced a decrease of CB_1_ receptor affinity with a concurrent loss of selectivity. CB_1_ receptor intrinsic activity of selected derivatives was evaluated through in vitro tests based on the determination of phosphorylated ERK 1/2 (P-ERK 1/2) expression in mouse neuroblastoma N1E-115 cell line. According to rimonabant, compounds **III*A*cX** and **III*B*cX** didn’t affect P-ERK expression in N1E-115 cells in the concentration range 1 nM-10µM; P-ERK 1/2 (% of vehicle): vehicle, 100 ÷ 10; rimonabant (1 µM): 110 ÷ 8; **III*A*cX** (1 µM): 111 ÷ 12; **III*B*cX** (1 µM): 95 ÷ 9. Furthermore, these compounds inhibited the P-ERK 1/2 expression up-regulation induced by the reference cannabinoid agonist WIN-55,212-2; vehicle: 185 ÷ 12; rimonabant (1 µM): 108 ÷ 7; **III*A*cX** (1 µM): 115 ÷ 13; **III*B*cX** (1 µM): 103 ÷ 9. In contrast, the myrtanil-based derivatives **III*I*cX** and **III*I*dX** enhanced P-ERK 1/2 expression in N1E-115 cells, highlighting CB_1_ receptor agonism profile for both compounds [61]. The pharmacological relevance of 1,4,5,6-tetrahydrobenzo(6,7)cyclohepta(1,2-*c*)pyrazole-based derivatives **III** emerged from the ability of antagonist NESS0327 to induce reduction of weight gain and food intake equivalent to that of rimonabant [64]. Furthermore, it was reported that the neutral antagonist profile exhibited by NESS0327 appeared important to avoid the side effects induced by rimonabant administration (i.e., suppression of the constitutive CB_1_ receptor activity in the ventral tegmental area and basolateral amygdala causing anxiety and reduced motivation for reward). Several patents testify to the pharmaceutical interest of NESS0327 [65,66,67,68]. Within this frame, the University of Queensland claimed method and agents for reducing general anesthetic induced neuroexcitation, attributing such side effect especially to neurosteroid general anesthetic agents as alfaxalone, even if the patent covers all general anesthetics in use [69]. In vitro electrophysiological analysis showed alfaxalone significantly decreased the amplitude of inhibitory synaptic currents (IPSC) on hypoglossal motor neurons, at a concentration ranging between 100 nM to 10 µM, consistent with increased in vivo motor activity and muscle twitching induced by alfaxalone. Treatment of hypoglossal motor neurons with NESS0327 was demonstrated to attenuate the effect of alfaxalone both on evoked than spontaneous IPSC amplitude. In vivo analysis conducted on Wistar rats highlighted administration of NESS0327 as a premedication prior to alfaxalone anesthesia significantly reduced muscle twitching during the induction and recovery phases of anesthesia. More importantly, premedication with NESS0327 had no detrimental effect on arterial O_2_ saturation or respiration rate during alfaxalone anesthesia [69].

### 4.2. 4,5-Dihydro-1H-benzo(2,3)oxepino(4,5-c)pyrazole-Based Derivatives (**VI**)

**Figure 15:**
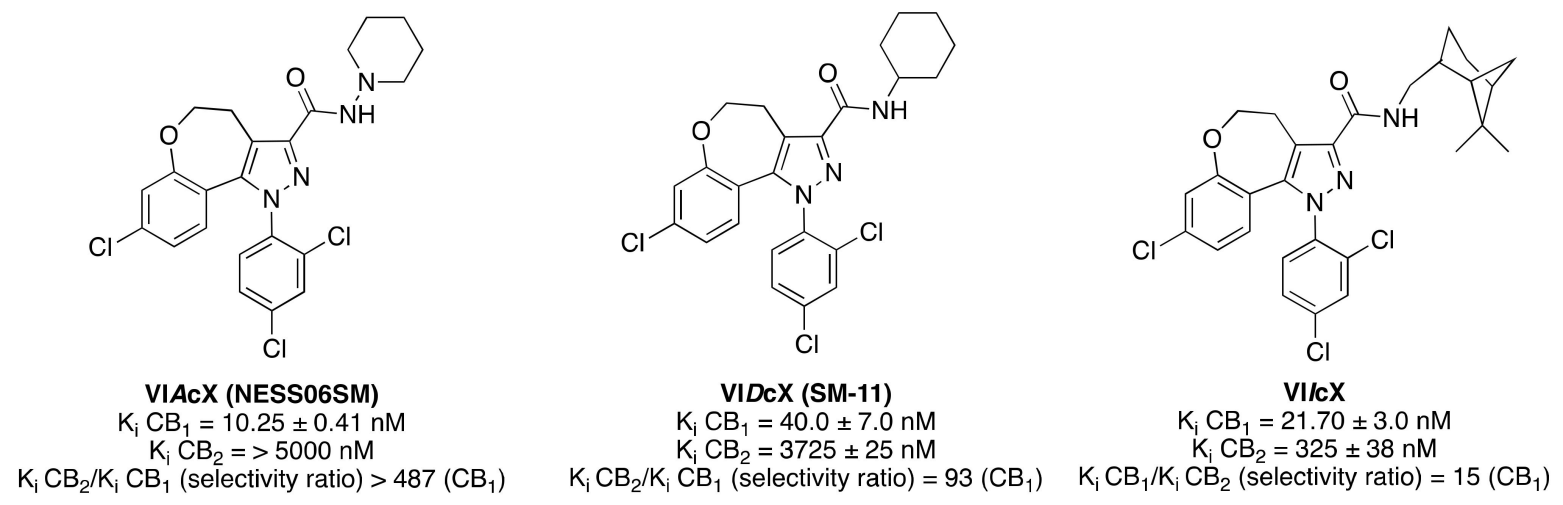
Structure of representative 4,5-dihydro-1*H*-benzo(2,3)oxepino(4,5-*c*)pyrazole-based derivatives (**VI**). Affinities at CB_1_ and CB_2_ receptors were assessed by competition of (^3^H)-CP 55,940 in mouse cerebellum membranes and in mouse spleen homogenates, respectively [73].

Endocannabinoids are orexigenic factors promoting appetite via CB_1_ receptor activation. This finding provided the rationale for the development of CB_1_ antagonist/inverse agonist rimonabant for obesity treatment and its metabolic complications. However, rimonabant side effects responsible for its withdrawal were principally related to both activities on SNC and to inverse agonism profile [70,71]. Therefore, new strategies have been proposed for the development of more safe anti-obesity agents, such as the identification of peripherally restricted CB_1_ receptor antagonists [71] as well as neutral CB_1_ receptor antagonists or CB_1_ allosteric modulators [72]. Within this frame, it was envisioned that bioisosteric modification of 1,4,5,6-tetrahydrobenzo(6,7)cyclohepta(1,2-*c*)pyrazole core (**III**) might offer new templates for CB_1_ receptor interaction. Thus, a series of 4,5-dihydro-1*H*-benzo(2,3)oxepino(4,5-*c*)pyrazoles (**VI**, Figure 2), containing an oxygen atom at position 6 in place of a methylene unit, were designed and synthesized. Such bioisosteric methylene/oxygen replacement might give access to CB_1_ ligands with increased polar surface area and decreased lipophilicity, which were considered critical parameters to influence the blood-brain permeability. Representative compounds are **VI*A*cX, VI*D*cX, VI*I*cX** (Figure 15), all exhibiting nanomolar/near nanomolar affinity for CB_1_ receptors [73]. Within this frame, a series of compounds sharing a 4,5-dihydro-1*H*-benzo(2,3)oxepino(4,5-*c*)pyrazole core, including **VI*A*cX**, were claimed by Cadila Healthcare Limited, but no binding data for cannabinoid receptors were presented [74]. However, the compounds of the invention were generally said, in the cAMP accumulation model, to antagonizes the WIN-55,212-2 inhibition of forskolin-induced cAMP accumulation in hCB_1_ CHO cells. Furthermore, **VI*A*cX**, and other representative compounds have been shown to reduce, in the sucrose solution intake rat model, the sucrose solution consumption [74,75]. In our hands, compound **VI*A*cX**, namely NESS06SM, emerged for its nanomolar CB_1_ receptor affinity and good selectivity with respect to CB_2_ one (K_i_ CB_1_ = 10.25 nM, K_i_ CB_2_ > 5000 nM). Evaluation of intrinsic activity carried out on NESS06SM highlighted a neutral antagonist profile both in (^35^S)GTPγS assay and in isolated organ assays (mouse vas deferens) [76]. In silico parameters (*c*LogP_OW_, *t*PSA, log BB), compared with **III*A*cX** (NESS0327) and other already known CB_1_ antagonists (i.e., rimonabant), suggested that NESS06SM exhibited sparing BBB permeability. Moreover, chronic treatment with NESS06SM resulted in the reduction of body weight and cardiovascular risk factor improvement in C57BL/6N diet-induced obesity (DIO) mice fed with a fat diet. Furthermore, in contrast to rimonabant, the chronic treatment of NESS06SM did not change mRNA expression of both monoaminergic transporter and neurotrophins, highly related to anxiety and mood disorders [76]. Interestingly, the co-treatment of NESS06SM has been shown to reduce food intake and weight gain, mitigate the side effects induced by chronic administration of the atypical antipsychotic olanzapine, without altering the positive effects of olanzapine on behavior [77].

As expected, evaluation of the intrinsic activity of **VI*D*cX**, namely SM-11, evidenced CB_1_ antagonist activity, both in in vitro test (P-ERK 1/2 expression in N1E-115 cells) and in isolated organs (mouse vas deferens). Behavioral studies highlighted that dose-dependently SM-11 decreased food intake in rats by 15–20%. Moreover, the i.v. administration of SM-11 fully and readily antagonized the effect of the agonist WIN-55,212-2 on the activity of ventral tegmental area (VTA) dopamine neurons projecting to the nucleus accumbens cells, confirming its antagonist profile. Furthermore, this data supported that SM-11 can lessen the hedonic aspect of food thus promoting bodyweight reduction [78].

### 4.3. 1,4,5,6-Tetrahydrothieno(2′,3′-6,7])cyclohepta(1,2-c)pyrazole-Based Derivatives (**IX**) and 1,4,5,6-Tetrahydrothieno(3′,2′-6,7)cyclohepta(1,2-c)pyrazole-Based Derivatives (**XII**)

A series of derivatives incorporating a 1,4,5,6-tetrahydrothieno(2′,3′-6,7)cyclohepta(1,2-*c*)pyrazole- and 1,4,5,6-tetrahydrothieno(3′,2′-6,7)cyclohepta(1,2-*c*)pyrazole-scaffolds **IX** and **XII**, respectively, (i.e., **IX*A*cX** or **XII*A*cX**, Figure 2) were claimed by Neuroscienze Pharmaness S.C.A.R.L. for cannabinoid receptor interaction, but no biological activity was presented [59].

### 4.4. 1,4,5,6-Tetrahydropyrazolo(3,4-c)pyrrolo(1,2-a)azepine-Based Derivatives (**XVII**)

**Figure 16:**
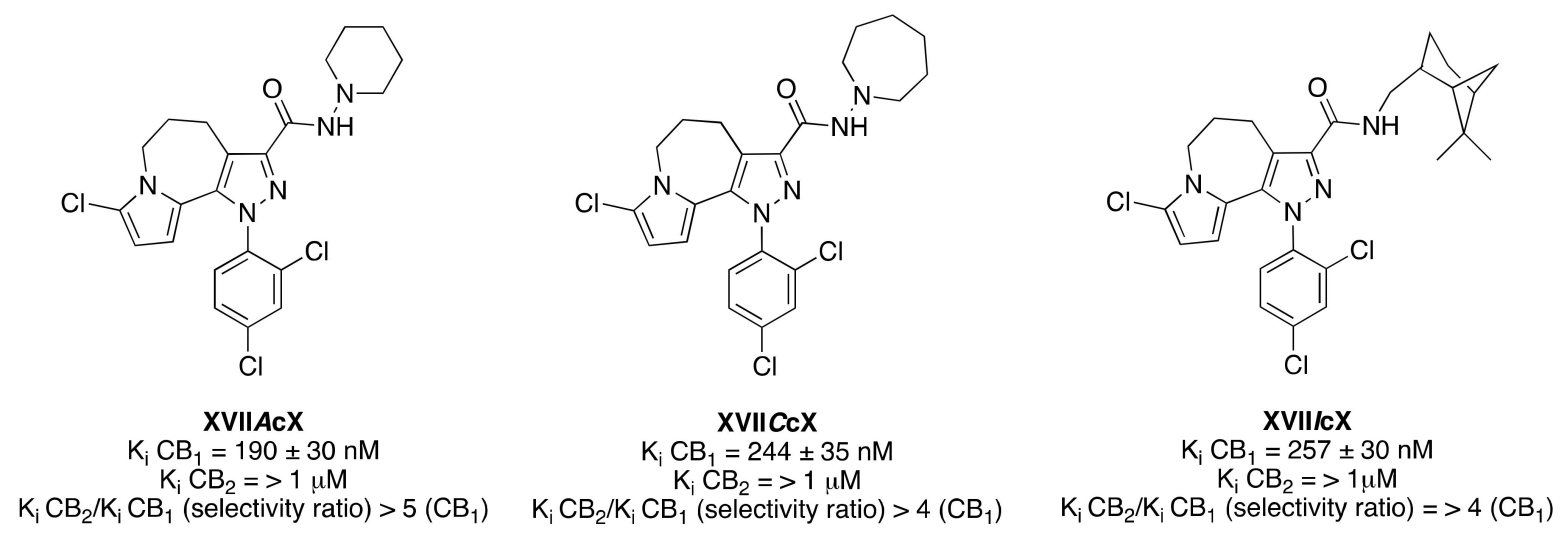
Structure of representative 1,4,5,6-tetrahydropyrazolo(3,4-*c*)pyrrolo(1,2-*a*)azepine-based derivatives (**XVII**). Affinities at CB_1_ and CB_2_ receptors were assessed by competition of (^3^H)-CP 55,940 in mouse whole-brain membranes and in CHO cell membranes transfected with hCB_2_ receptors [56].

A series of 1,4,5,6-tetrahydropyrazolo(3,4-*c*)pyrrolo(1,2-*a*)azepine-based derivatives (**XVII**, Figure 2) has been investigated for cannabinoid receptor interaction [56]. Representative compounds are depicted in Figure 16, all devoid of affinity for CB_2_ receptors and endowed of negligible affinity for CB_1_ receptors.

## 5. (5,8)-Condensed Pyrazole Derivatives

### 4,5,6,7-Tetrahydro-1H-benzo(7,8)cyclooct(1,2-c)pyrazole-Based Derivatives (**IV**)

**Figure 17:**
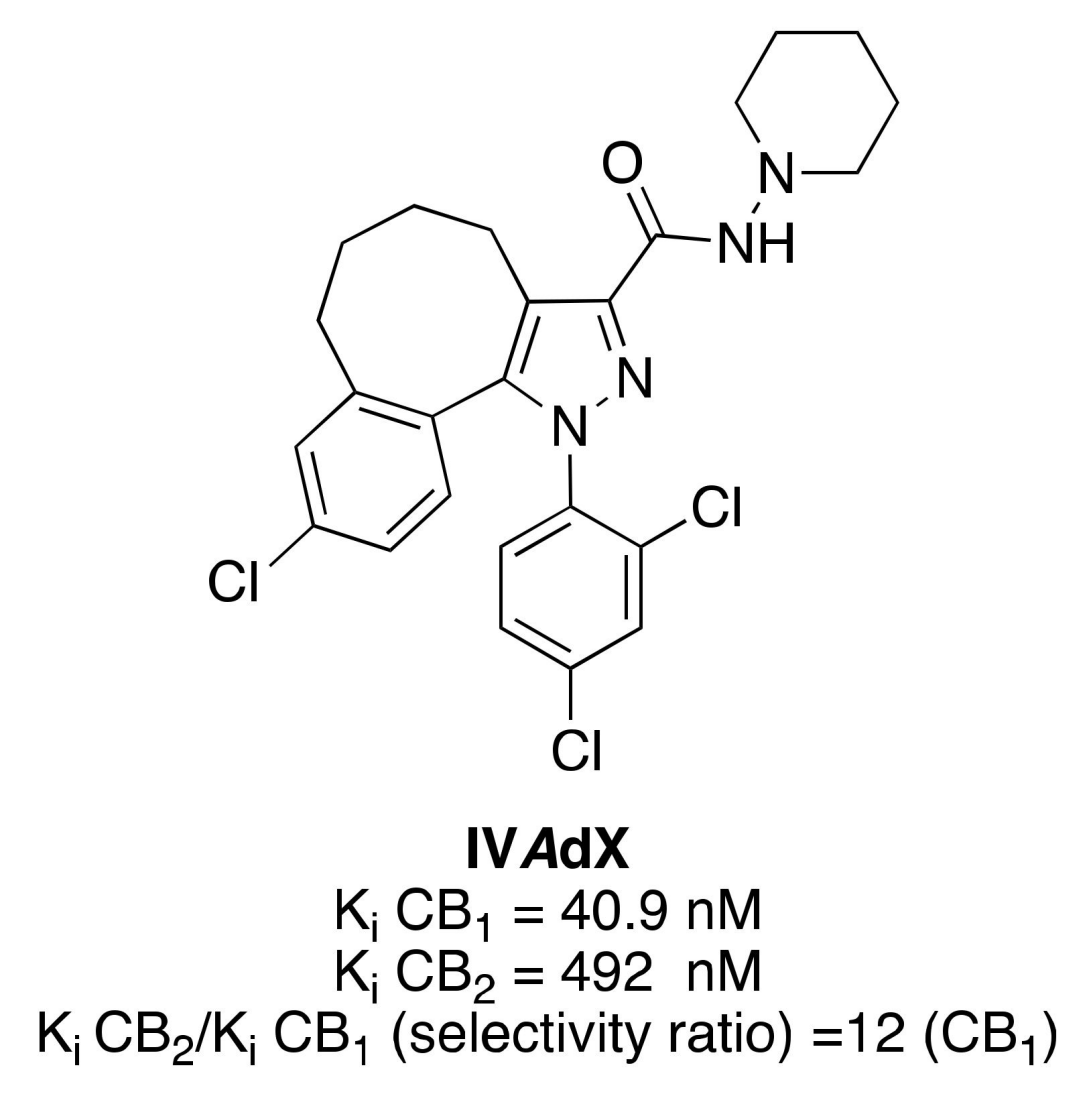
Structure of 4,5,6,7-tetrahydro-1*H*-benzo(7,8])cycloocta(1,2-*c*)pyrazole derivative **IV*A*dX**. Affinities at CB_1_ and CB_2_ receptors were assessed by competition of (^3^H)-CP 55,940 in HEK-293 and CHO-K1 cell membranes, respectively [63].

Zhang et al. described the synthesis and biological evaluation of a series of conformationally constrained analogs of rimonabant [63]. Within this frame, they synthesized both compound **III*A*cX** from our lab and **IV*A*dX** (Figure 17) which is the only pyrazole-based tricyclic compound known belonging to (5,8)-condensed derivatives, featuring the 4,5,6,7-tetrahydro-1*H*-benzo(7,8)cycloocta(1,2-*c*)pyrazole core. The compound, endowed of lower affinity for CB_2_ than CB_1_ receptors, exhibited a similar trend with respect to homologous compounds **II*A*bX** and **III*A*cX**. However, increasing the carbocyclic central ring size by one methylene unit, i.e., from (5,7)- to (5,8)-condensed pyrazole derivatives, involved a marked loss of affinity for CB_1_ receptors.

## 6. Miscellaneous Derivatives

**Figure 18:**
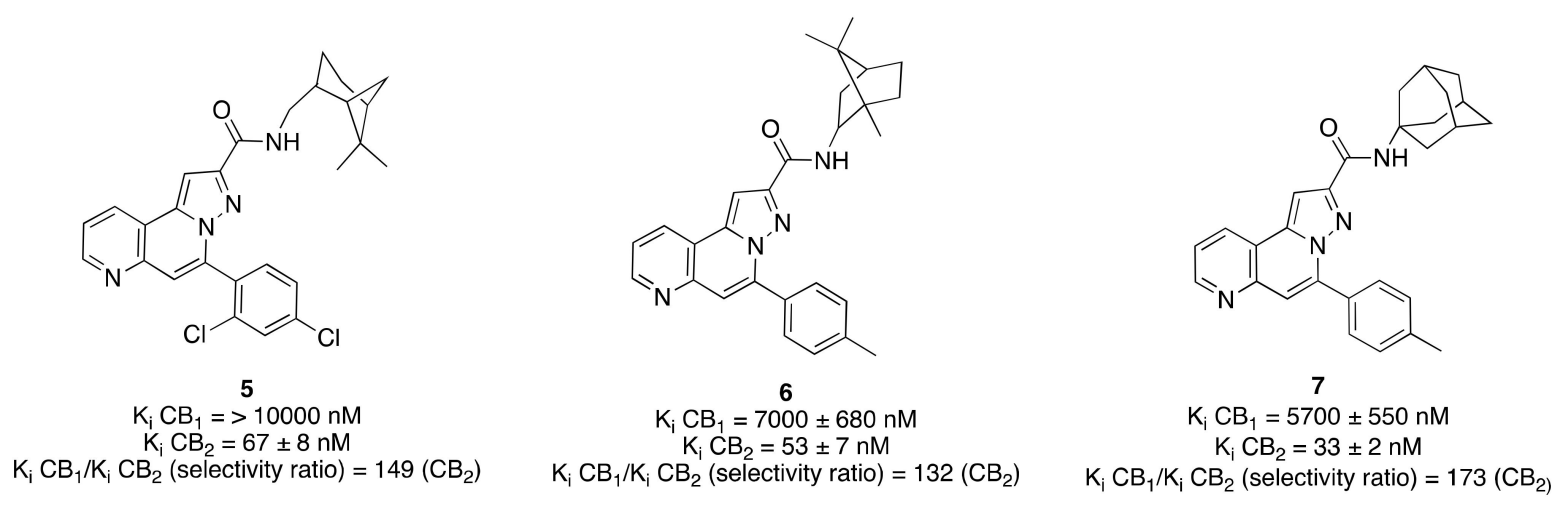
Structure of representative pyrazolo(5,1-*f*)(1,6)naphthyridine-based derivatives (**5**–**7**). Affinities at CB_1_ and CB_2_ receptors were assessed by competition of (^3^H)-CP 55,940 in transfected human CB_1_ and CB_2_ CHO cells [79].

Other pyrazole-based tricyclic derivatives, featuring the pyrazolo(5,1-*f*)(1,6)naphthyridine core (Figure 18), were investigated for cannabinoid receptor interaction [79].

**Scheme 2:**
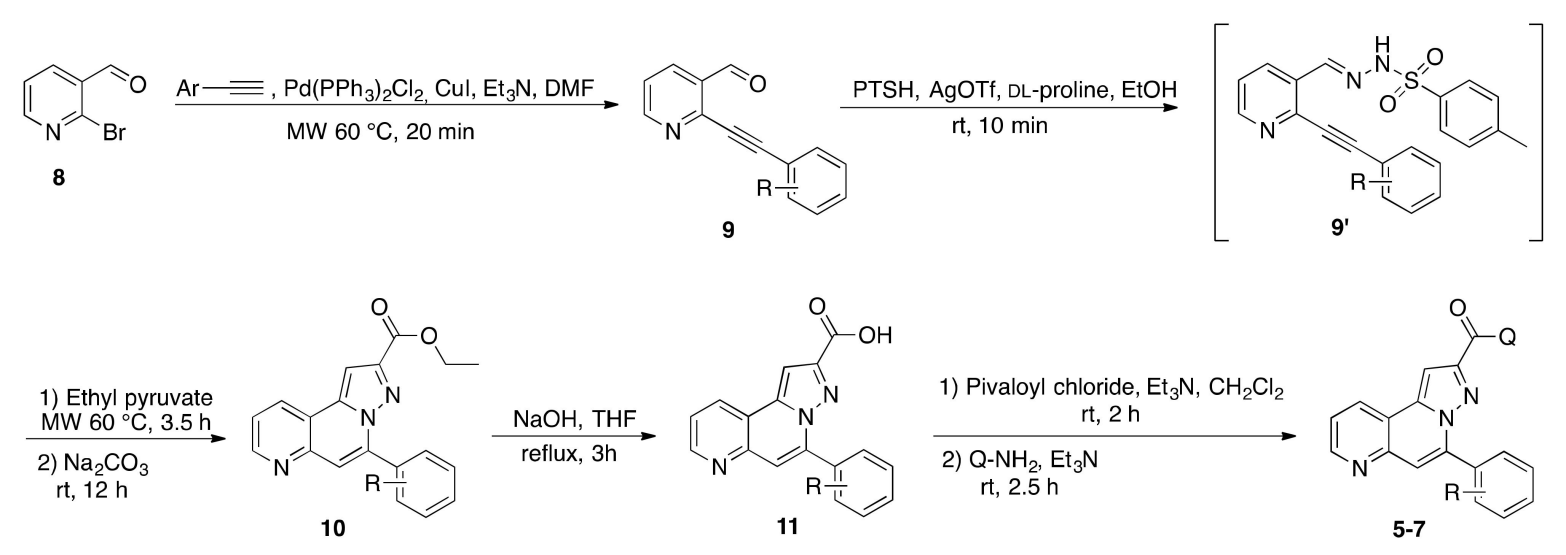
General synthetic route for the preparation of pyrazolo(5,1-*f*)(1,6)naphthyridine derivatives **5–7**.

The pyrazolo(5,1-*f*)(1,6)naphthyridine derivatives were synthesized making use of AgOTf and proline-cocatalyzed multicomponent methodology (Scheme 2), starting from the appropriate *o*-alkynylaldehydes **9**, *p*-toluenesulfonyl hydrazide (PTSH) and ethyl pyruvate, to gave key pyrazole-esters **10** which, hydrolyzed with NaOH, afforded the corresponding acids **11**. Target compounds were synthesized by condensation of acids **11**, previously activated with pivaloyl chloride, with the appropriate amines. The *o*-alkynylaldehydes **9** were obtained from 2-bromonicotinaldehyde **8** and appropriate alkynes which were reacted under the conventional Sonogashira conditions.

Compounds **5–7** exhibited affinity levels for CB_2_ receptors in the near nM range (K_i_: 33–67 nM) with a good degree of selectivity for CB_2_ receptors compared to CB_1_. According to in vitro assays based on the effects of forskolin-stimulated cAMP levels in human CB_2_ CHO cells, compounds **5–7** exhibited antagonist/inverse agonist properties [79].

## 7. Conclusions

In the last decades, the CB_1_ antagonist/inverse agonist rimonabant has been considered an extremely valuable lead for the design of new ligands for CB receptors interaction, with potential therapeutic value. In the context of this review, the introduction of structural constraints in rimonabant, the use of medicinal chemistry approaches as homologation or bioisosterism, gave access to several compounds most of which belong to (5,5), (5,6) and (5,7)-condensed tricyclic pyrazole derivatives. Here we have summarized the extensive SAR studies carried out on such compounds, allowing us to identify different ligands endowed with high affinity and selectivity for CB_1_ or CB_2_ receptors. In particular, the pharmacological relevance of 1,4-dihydroindeno(1,2-*c*)pyrazole-based **I** compounds, belonging to (5,5) tricyclic pyrazole derivatives, emerged from the significant anti-nociceptive activity of the potent and selective CB_2_ agonist **I*D*hX**, by alleviating both mechanical allodynia and thermal hyperalgesia in Spared Nerve Injury (SNI) neuropathic mice. The interest of ligands belonging to (5,5) tricyclic pyrazole derivatives emerged also for their potential as anti-glaucoma agents, for the ability to reduce intraocular pressure (IOP). Compound **X*I*hZ**, featuring the 1,4-dihydrothieno(3′,2′-4,5)cyclopenta(1,2-*c*)pyrazole core and exhibiting high and mixed CB_1_/CB_2_ receptor affinities, effectively reduced intraocular pressure in the animal model of old DBA/2J mice, providing a potential alternative to the use of WIN-55,212-2 and smoking marijuana, known for their IOP lowering properties. SAR study pointed out the relevance of ligands belonging to (5,7) tricyclic pyrazole derivatives, for their anti-obesity potential. In this frame, the selective CB_1_ ligand **VI*A*cX**, featuring the 4,5-dihydro-1*H*-benzo(2,3)oxepino(4,5-*c*)pyrazole core, exhibiting neutral antagonist profile, effectively reduced body weight with the improvement of cardiovascular risk factor in C57BL/6N diet-induced obesity (DIO) mice fed with fat diet. Preliminary data evidenced the potentiality of compound **VI*A*cX** in the treatment of obesity with a more safe profile with respect to the antagonist/inverse agonist rimonabant.

## Figures and Tables

**Table 1:** Intraocular pressure (IOP) variation in old DBA/2J mice after administration of compounds **X*I*hZ, X*N*hZ, X*H*hX** and WIN-55,212-2 as reference compounds. The results are expressed as percent decrease of the IOP with respect to the animal basal IOP value [41].

	Intraocular Pressure Decrease (%)									
Time of Administration	Carrier		X*I*hZ		X*N*hZ		X*H*hX		WIN-55,212-2	
(Minutes)	20 µL	40 µL	50 µg	100 µg	50 µg	100 µg	50 µg	100 µg	50 µg	100 µg
30	0.1	0.3	15.9	23.9	2.9	22.2	3.0	16.6	4.3	23.1
60	−0.1	0.0	15.0	22.8	2.2	22.4	2.0	14.0	3.0	21.3
90	0.2	−0.2	12.6	19.6	0.8	25.0	0.5	11.2	2.0	18.1
120	0.0	0.0	11.7	21.7	1.5	16.7	11.7	21.7	1.9	13.2
